# Effects of Aging on Correlation of Striated Esophageal and Pharyngeal Deglutitive Motor Function

**DOI:** 10.1155/grp/9920130

**Published:** 2026-06-10

**Authors:** Mark Kern, Francis Edeani, Ling Mei, Patrick Sanvanson, Reza Shaker

**Affiliations:** ^1^ Division of Gastroenterology and Hepatology, Medical College of Wisconsin, Milwaukee, Wisconsin, USA, mcw.edu

**Keywords:** aging, manometry, peristalsis, pharynx, striated muscle esophagus

## Abstract

**Introduction:**

Effects of aging on the relationship of striated esophagus (StEso) motor function with pharyngeal deglutitive biomechanics has not been systematically studied.

**Objective:**

The aim of this study is to characterize the effects of aging on the correlation of deglutitive StEso with pharyngeal function.

**Materials and Methods:**

We studied 33 healthy subjects. Fifteen elderly (age: 75 ± 7 years, 8 female) and 18 nonelderly (age: 50 ± 14 years, 9 F) were evaluated using high resolution manometry/impedance. Nine elderly (73 ± 7 years, 5 F) and eight young (24 ± 3 years, 4 F) were further evaluated by digital videofluoroscopy.

**Results and Discussion:**

Duration of StEso excursion averaged 2.56 ± 0.65 s in elderly and 2.33 ± 0.41 in young. We identified four periods in StEso motor function during deglutitive excursion: (a) anterosuperior ascent without bolus/peristaltic activity, (b) nonperistaltic bolus receiving function at apogee of StEso excursion during UES opening and pharyngeal peristalsis, (c) peristaltic bolus transport as StEso descends, (d) continued peristalsis in resting position. Apart from the final period described, the periods are significantly different in the elderly compared with the young subjects (*p* < 0.04). Furthermore, the interaction of striated esophageal flow dynamics and peristaltic contractile vigor showed that a significant correlation (*r* = −0.71, *p* = 0.0009) was found when comparing the average bolus injection length to the average SECI in young subjects. This correlation was muted in the elderly and did not reach statistical significance (*r* = −0.41, *p* = 0.13).

**Conclusions:**

Sequencing of StEso/pharyngeal deglutitive motor function is preserved in the elderly. The differences observed in early stages of spatiotemporal mapping of StEso deglutitive motor function when comparing healthy elderly to young populations may be due to age‐related suprahyoid muscle weakness since the affected periods are those associated with the active StEso ascent and descent. Like young individuals, elderly pressure signatures currently attributed to the StEso deglutitive motor activity do not represent the entirety of StEso peristalsis.

## 1. Introduction

Although the deglutitive function of the pharynx and esophagus individually has been the subject of many studies, the aspect of these organs′ motor function that is implicated in the transfer of the swallowed bolus from the pharynx into the esophagus has only recently received investigative attention [1, 2]. Investigating this aspect of pharyngeal and esophageal motor function is complex due to the swallow‐induced axial motion of the proximal esophagus along with the upper esophageal sphincter (UES) and shortening of the pharynx [3], which necessitates multimodal techniques such as concurrent manometry, impedance recording and fluoroscopy. As such, despite the high prevalence of swallowing abnormalities including among the elderly, the potential role of this bolus transfer aspect of pharyngeal and esophageal motor function in the pathophysiology of dysphagia has not been systematically evaluated. Understanding this aspect of swallowing among the elderly becomes more significant as both organs, which are comprised of striated muscle, are subject to age‐induced deterioration potentially negatively affecting the safe transfer of the bolus from the pharynx to the proximal striated esophagus (StEso). Recent studies of pharyngeal and esophageal motor function involved in bolus transfer from the pharynx revealed four periods.

Period 1 begins with the onset of StEso and laryngeal anterosuperior excursion and ends with the onset of UES opening wherein the StEso does not exhibit any peristaltic activity and remains devoid of the swallowed barium bolus. Period 2 starts with the onset of UES opening and trans‐sphincteric pharyngo‐esophageal bolus transit spanning the duration of UES opening and ending with complete UES closure. This period occurs at or near the time of the maximum StEso, UES, and laryngeal excursion when the pharyngeal bolus is forcefully injected through the open UES into the StEso by posterior tongue thrust and hypopharyngeal peristalsis. At the end of Period 2 the tail of the bolus is at the level of closed UES as the rest of the bolus spreads within the StEso. Like Period 1, the StEso does not exhibit any peristaltic activity in Period 2. Period 3 begins with the complete closure of the UES and initiation of the StEso peristalsis actively transporting the bolus caudally. This period occurs during the StEso, UES, and laryngeal descent toward resting position. Onset of StEso descent is tightly related to the UES closure and initiation of StEso peristalsis. This period ends with the arrival of the StEso back to its resting position. Period 4 begins with the end of StEso descent and eventual arrival to resting position and includes the continuation of the distal progression of the peristaltic wave. This period ends as determined by the concurrently recorded high‐resolution manometry (HRM), with the arrival of StEso peristalsis at the esophageal transition zone, which manometrically demarcates the distal end of the StEso [1, 2]. The benefits of recognizing these distinct periods during swallowing are twofold; first is the physiologic insight into the symbiotic relationship between the pharynx, UES, and StEso during deglutition; second is the important insight informing interpretation of pharyngo‐esophageal manometric isocontour data.

Concurrent with the four phases of UES and StEso kinematics and dynamics described above is the resulting effects on the display and interpretation of HRM isocontour pharyngo‐esophageal pressure recordings. Since the HRM catheter is largely stationary during the intervals of pharyngeal and initial StEso deglutitive peristalsis, as the pharynx, UES, and StEso make their ascending and descending paths during swallowing, there are times in the recorded manometric isocontour when pressure sites in the resting pharynx are covered by the rising and falling UES and StEso. Although this phenomenon has been previously reported [4], the manometric ramifications have been not been systematically evaluated and interpreted until recently [2]. Given the conventional, historically accepted interpretation of pharyngeal manometric data [5–10], proximal esophageal peristaltic wave pressures may be inadvertently incorporated into “pharyngeal” peristaltic contractile activity metrics derived from manometric recordings. Since clinical manometry is commonly not performed concurrently with fluoroscopy, falsely attributing pressure activity to the pharynx in the isocontour display is a potential diagnostic problem. Compounding the difficulty of accurately interpreting pharyngo‐esophageal manometry is the false attribution as pharyngeal peristalsis of the pseudo‐peristaltic pressure wave generated by the closed, contracting UES as it traverses over the pharyngeal sensors during its descent to resting position.

Furthermore, metrics of the dynamic interplay between the propulsive forces generated by deglutitive pharyngeal biomechanics and the receptive as well as contractile properties of the StEso have not been systematically evaluated. In simplest terms, this interplay is characterized by a fundamental question: Does the distance a liquid bolus is propelled into the esophagus correlate with the contractile vigor of the subsequent striated muscle peristaltic contractility? Moreover, is this interaction affected by aging? Previous studies have shown the effects of aging on pharyngeal deglutitive biomechanics to the extent that aging affects hyolaryngeal movement [11–19], UES opening [17, 20–23], airway protection [24, 25] as well as the impact of exercise on the pharyngeal swallowing muscles [19, 26–28]. Although there is a robust literature regarding pharyngeal deglutitive biomechanics, research exploring the interaction between pharyngeal deglutitive function and StEso function is sparse. Recent reports of pilot data from our lab have shown correlation between the amount of swallow‐induced stretch of the proximal esophagus and increased SECI in human [29] and in feline models [30]. A more direct measure of the proposed dynamic interaction between pharyngeal propulsion and SECI is the recently described bolus injection length (BIL) [31], namely, the preperistaltic projection length of a swallowed liquid bolus into the StEso prior to development of primary peristalsis. To gauge the interaction of pharyngeal dynamics and StEso peristaltic contractility, the BIL may affect or be correlated with a direct measure of StEso contractile vigor, namely the striated esophageal contractile integral (SECI). Contractile integral measurements are a product of the isocontour representation of esophageal and pharyngeal peristaltic pressures and have been documented in measurements of proximal [32–36] and distal [31, 37–39] esophageal as well as pharyngeal [6, 7, 26, 28, 40–44] contractile vigor.

To address these issues, the aims of the present study were to characterize and quantify the effects of aging on: (1) the correlation of the StEso deglutitive function with pharyngeal phase swallowing and (2) the inter‐relationship between nonperistaltic propulsion of the pharyngeal bolus and SECI.

## 2. Material and Methods

Studies were approved by the Internal Review Board of the Human Research Protection Program at the Medical College of Wisconsin (Milwaukee, Wisconsin) and all subjects gave written informed consent prior to their studies.

A total of 33 healthy subjects were studied. Fifteen elderly subjects (age: 75 ± 7 years, 8 female) and 18 nonelderly (age: 50 ± 14 years, 9 female) were evaluated using high resolution manometry and impedance (Manoscan system, Medtronics, Minneapolis, Minnesota). All subjects were studied in a seated, upright position while swallowing 5 mL saline solution (three repetitions). Among these subjects, nine elderly healthy volunteers (73 ± 7 years, 5 F) and eight young healthy volunteers (24 ± 3 years, 4 F) were evaluated by digital videofluoroscopy (30 frame/s) during swallowing 5 mL 40% *W*/*V* liquid barium concurrent with high resolution manometry and impedance. All subjects with concurrent manometry and fluoroscopy were studied in a seated, upright position with the volunteer′s head in a neutral position. We used laryngeal excursion as a surrogate for StEso excursion because of their anatomic connection.

Videofluoroscopic recordings were made in sagittal view at 90 keV, using a 9‐in. image intensifier and appropriate collimation. We recorded fluoroscopic movies of 5‐mL thin liquid barium swallows, each repeated three times. Subjects were verbally cued to perform three swallows of 5 mL thin barium solution at 20‐s intervals. Barium boluses were slowly injected in the oral cavity by a syringe and swallowed as a single bolus. Fluoroscopic images were centered on the pharyngoesophageal junction to clearly visualize the pharynx, UES, proximal esophagus, and larynx. Images were digitally stored for subsequent stop‐action kinematic video analysis using the ImageJ software package [45].

Pharyngeal and proximal esophageal pressures were recorded simultaneously using a high‐resolution manometric catheter positioned trans nasally to traverse the pharynx, UES, and proximal esophagus and part of the remaining esophagus. The manometric probe and its associated computerized recording and analysis system (ManoScan and ManoView Systems; Given Imaging, Duluth, Georgia) store pressure data from 36 pressure sensors (1‐cm sensor spacing), display manometric information in topographic or line graph formats, and provide postacquisition analytic tools for parameterization of temporal and spatial pressure data. Digital fluoroscopic images were captured concurrently with the manometric data.

Fluoroscopic images were analyzed regarding the timing of several important kinematic deglutitive events including onset of laryngeal ascent, laryngeal peak movement, onset of laryngeal descent, laryngeal rest position, UES opening, UES closure, and onset of pharyngeal postdeglutitive opening and epiglottal movement. The onset of laryngeal ascent was defined as the first frame in the videofluoroscopic recording of a swallow associated with the initial anterosuperior excursion of the anterior superior portion of the subglottic air column from rest. Laryngeal peak movement was defined as the maximal anterosuperior deglutitive excursion of the anterior superior portion of the subglottic air column. The onset of laryngeal descent was defined as the first videofluoroscopic frame associated with the posterior–inferior return of the anterior superior portion of the subglottic air column back to its resting position. Return of the larynx to resting position was defined as the videofluoroscopic frame associated with return of the anterior superior portion of the subglottic air column to rest. Onset of deglutitive UES opening was defined as the videofluoroscopic frame when the barium contrast initially is seen separating the anterior and posterior walls of the pharyngoesophageal segment. UES closure was defined as the videofluoroscopic frame when the barium contrast no longer separates the anterior and posterior walls of the pharyngoesophageal segment. The first fluoroscopic evidence of any postdeglutitive pharyngeal opening was defined as the first postdeglutitive videofluoroscopic frame wherein air can be seen anywhere in the pharynx. Onset of postdeglutitive epiglottic opening was defined as the first postdeglutitive videofluoroscopic frame wherein the epiglottis was seen to move from its down‐folded deglutitive position. Full postdeglutitive epiglottic opening was defined as the videofluoroscopic frame wherein the epiglottis has returned to its resting position.

Like the technique for measuring laryngeal position described above, the speed of laryngeal descent was also measured from the fluoroscopic images of the anterior/superior portion of the subglottic air column. Using the distance measuring feature of the ImageJ software [45], the distance traveled by the anterior/superior portion of the subglottic air column was measured from the moment of initial laryngeal descent to rest along with the number of frames required to traverse the measured distance, thereby yielding the average velocity of laryngeal descent.

The velocity of peristalsis was measured from both the fluoroscopic images as well as the HRM recordings. From the fluoroscopic images, the distance traveled by the tail of the barium bolus after UES closure was measured marking the spatial location of the tail of the barium bolus immediately after UES closure and advancing the video fluoroscopic images to the frame just before the tail of the bolus left the field of view and marking this spatial location. With these two markers, the distance traveled by the bolus tail is known along with the number of frames between those markings yielding the average peristaltic wave speed after UES closure. From the manometric data, StEso peristaltic wave speed was calculated in two ways. Using a functional feature of the ManoView software, the slope of the 20‐mmHg isobar in the topographic display of pharyngo‐esophageal pressure was measured. Additionally, the ManoView line chart representation of the 1‐cm spaced manometric recording sites was used to track the abrupt rise of pressure due to peristalsis across the pressure sites in the UES and StEso. By noting the time of pressure upstroke due to peristalsis across the pharyngo‐esophageal pressure sites, the average velocity was calculated.

Statistical analysis included descriptive statistics, distribution analyses, hypothesis testing of mean and median values. Data are presented as mean ± standard deviation if normally distributed or as median with upper and lower 5th percentile if not normally distributed. Interobserver reproducibility was tested using intraclass correlation analysis.

## 3. Results

Participants tolerated the studies without untoward events.

**Figure 1:**
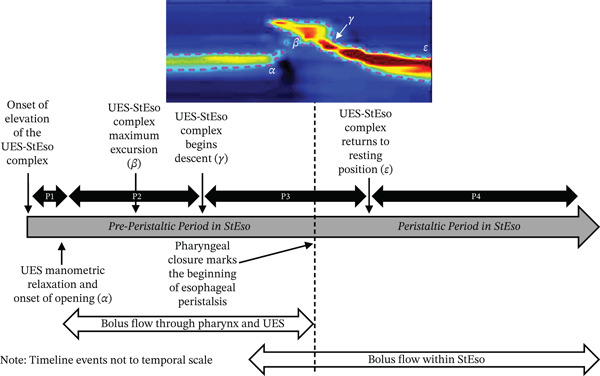
Schematic diagram showing the correlation of striated esophageal (StEso) and pharyngeal motor events observed by concurrent digital videofluoroscopy and high‐resolution manometry. These events include the following: P1 = anterosuperior ascent of the StEso and UES without bolus or peristaltic activity. P2 = UES opening and propulsion of the bolus associated with pharyngeal peristalsis into the StEso without peristaltic activity at the apogee of StEso excursion during UES opening and pharyngeal peristalsis. P3 = peristaltic/bolus transport as StEso descends. P4 = continued StEso peristalsis in resting position.

Figure 1 shows schematically the correlation of the StEso, and pharyngeal motor events observed by concurrent digital videofluoroscopy and high‐resolution impedance manometry. These events include:

• P1 = anterosuperior ascent of the StEso and UES without bolus or peristaltic activity.
• P2 = UES opening and propulsion of the bolus associated with pharyngeal peristalsis into the StEso without peristaltic activity at the apogee of StEso excursion during UES opening and pharyngeal peristalsis.
• P3 = peristaltic/bolus transport as StEso descends.
• P4 = continued StEso peristalsis in resting position.

Comparison of the duration of each period between young and elderly is shown in Table 1. UES opening duration coincided with bolus moving into the StEso (bolus receiving period) during which bolus was injected into the StEso prior to start of peristalsis. This period was followed by peristalsis, transporting the bolus caudally. StEso peristalsis occurred immediately following UES closure and progressed as the StEso descended and continued when it reached its resting position. As seen in Table 1, although the increase in total duration of StEso excursion in elderly did not reach statistical significance, duration of P1, P2, and P3 was found to be significantly different in the elderly compared with the young subjects (*p* < 0.04). However, P1 showed a significant decrease in the elderly, whereas P2 and P3 showed significant increases possibly explaining the lack of significant statistical difference in the total duration value.

**Table 1:** Duration in seconds of the four periods of StEso motor function.

	Pre‐receiving period (P1)	Receiving period (P2)	Peristaltic period		Total excursion period
			UES closure to larynx at rest (P3)	During larynx at rest (P4)	
Elderly	0.12 ± 0.03^∗^	0.59 ± 0.12^∗^	0.75 ± 0.12^∗^	1.10 ± 0.30	2.56 ± 0.65
Young	0.19 ± 0.03	0.45 ± 0.13	0.60 ± 0.06	1.09 ± 0.27	2.33 ± 0.41

^∗^ *p* < 0.04, elderly versus young.

**Figure 2:**
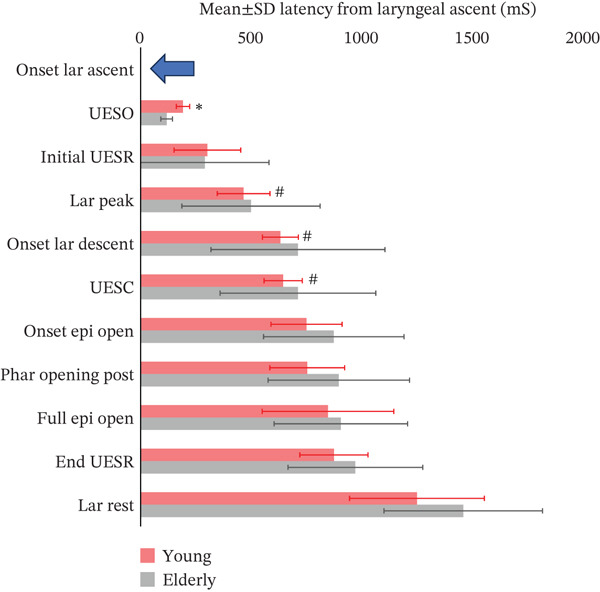
Comparison of the sequence of pharyngeal kinematic and biomechanical events associated with bolus transport into the StEso between young and elderly. Average for latency from the onset deglutitive laryngeal movement are longer for the elderly compared with the young but statistically significant differences were realized for UESO ( ^∗^ *p* < 0.001).

Comparison of the sequence of pharyngeal kinematic and biomechanical events associated with bolus transport into the StEso between young and elderly is shown in Figure 2. As seen, in general the average for latency from the onset of deglutitive laryngeal movement is longer for the elderly compared with the young, but statistically significant differences were realized for UESO ( ^∗^ *p* < 0.001). Of note, significant differences in variance were more common than significant groupwise differences in average latency values. As seen in Figure 2, latency to laryngeal peak deglutitive excursion, onset of laryngeal descent, and UES closure all showed significantly different variances (*p* < 0.03, F‐test) when comparing elderly to young values.

As seen in Table 2, the order of occurrence of pharyngeal phase deglutitive biomechanical events relevant to StEso motor function measured across all swallows showed many subtly different combinations and permutations. In all observed sequences as seen previously [1], UES opening and the peak of StEso/laryngeal excursion always were the first events following the onset of laryngeal ascent, whereas complete UES closure and beginning of StEso peristalsis always occurred at the onset of the larynx/StEso descent or shortly thereafter. Although there was considerable overlap in the timing of the deglutitive events among studied individuals, UES closure signifying the completion of pharyngeal peristalsis and the beginning of StEso peristalsis occurred during laryngeal and StEso descent before they returned to their resting position.

**Table 2:** Examples of the most prevalent permutations for sequences of pharyngeal phase deglutitive biomechanical events and StEso motor function.

Event 1	Event 2	Event 3	Event 4	Event 5	Event 6	Event 7	Event 8	Event 9	Frequency	
									Elderly	Young
OLA	USO	LPM	OLD	USC	PPO and OEO		FEO	LRP	15	17
OLA	USO	LPM	OLD	USC	PPO	OEO	FEO	LRP	11	13
OLA	USO	LPM	OLD and USC		PPO and OEO		FEO	LRP	7	8
OLA	USO	LPM	USC	OLD	PPO	OEO	FEO	LRP	7	8
OLA	USO	LPM	OLD and USC		PPO	OEO	FEO	LRP	7	8
OLA	USO	LPM	OLD	USC	OEO	PPO	FEO	LRP	7	4
OLA	USO	LPM	USC	OLD	PPO and OEO		FEO	LRP	7	4
OLA	USO	LPM	OLD	PPO and OEO		USC	FEO	LRP	4	4

Abbreviations: FEO, full postdeglutitive epiglottic opening; LPM, peak of deglutitive laryngeal anterosuperior movement; LRP, return of larynx to resting position; OEO, onset of postdeglutitive epiglottic opening; OLA, onset of deglutitive laryngeal ascent; OLD, onset of laryngeal descent; PPO, first fluoroscopic evidence of air during postdeglutitive pharyngeal opening; USC, UES closure; USO, onset of deglutitive UES opening.

**Figure 3:**
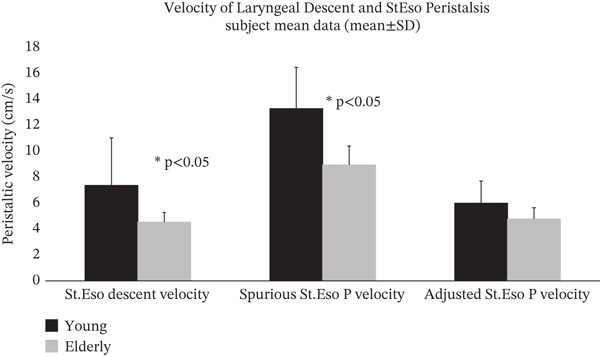
Average velocity of StEso peristalsis in Period 3 as the organ is descending toward its resting position observed in HRM isocontour. Spurious velocities (11.61 ± 3.63 cm/s) far exceed that observed in manometric recordings in Period 4 in this study (5.38 ± 2.18 cm/s) when the StEso was in its resting position. The mean laryngeal descent velocity was measured at 7.4 ± 3.7 cm/s in young subjects and 4.5 ± 0.7 cm/s in elderly subjects ( ^∗^ *p* = 0.0295). The hashed bars show the result of subtraction of StEso descent velocity from the recorded/observed velocity representing the real velocity of StEso peristalsis. There was no significant difference in the adjusted StEso peristaltic velocity when comparing young to elderly subjects.

We evaluated St. Eso peristaltic wave velocity as manifested in Period 3 in both groups using HRM. StEso peristaltic velocity values reported in the literature have been primarily derived from HRM using either topographic or channel‐wise line graph analyses. StEso peristaltic wave speed was calculated by measuring the slope of the 20‐mmHg isobar in the topographic display. The average velocity of StEso peristalsis in period 3 as the organ is descending toward its resting position observed in HRM isocontour is depicted in Figure 3. Note that StEso peristaltic wave in this period is recorded by HRM sensors that are in the pharynx at rest, but during ascent the StEso has climbed onto them and, as such, the StEso peristaltic pressures will be reflected in the most distal part of the pharyngeal peristalsis isocontour between the time of UES closure on fluoroscopy and the end of UES manometric relaxation identified on HRM. These spurious velocities (11.61 ± 3.63 cm/s) far exceed that observed in manometric recordings in period 4 in this study (5.38 ± 2.18 cm/s) when the StEso was in its resting position as well as those values reported in the literature [46–48]. To further evaluate this discrepancy, since the speed of peristaltic wave in the descending/moving StEso in Period 3 is measured using stationary reference points on the intraluminal pressure sensor, we assumed the measured speed represented the sum of the actual velocity of the peristalsis and the velocity of the StEso/laryngeal descent. As seen in Figure 3, the mean laryngeal descent velocity was measured at 7.4 ± 3.7 cm/s in young subjects and 4.5 ± 0.7 cm/s in elderly subjects ( ^∗^ *p* = 0.0295, Mann–Whitney *U* Test). The hashed bars in Figure 3 show the result of subtraction of StEso descent velocity from the recorded/observed velocity representing the real velocity of StEso peristalsis in Period 3. There was no significant difference in the adjusted StEso peristaltic velocity when comparing young to elderly subjects. There was no significant difference between the peristaltic wave speeds calculated using the two techniques described in the Methods section.

**Figure 4:**
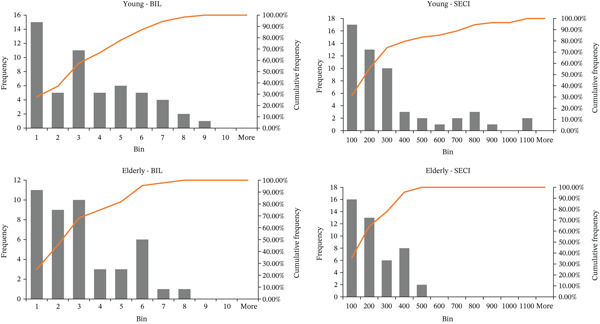
Frequency and cumulative distributions of BIL and SECI in elderly study population showed nonnormal distributions slightly skewed right. These distributions show that there are fewer observations of larger SECI and BIL values in the elderly compared with young.

As seen in Figure 4, frequency and cumulative distributions of BIL and SECI in the elderly study population showed nonnormal distributions slightly skewed right. These distributions show that there are fewer observations of larger SECI and BIL values in the elderly compared with young data. Descriptive statistics are shown in Table 3. The disparities in distribution did not manifest significant differences when comparing young to elderly BIL and SECI values; however, the lower values for BIL and SECI are suggestive of diminished striated muscle performance in the tested elderly compared with young.

**Table 3:** Descriptive statistics for frequency and cumulative distributions of BIL and SECI elderly compared with young data.

	BIL (cm)					SECI (mmHg‐cm‐s)				
	Mean	SD	Median	5th %	95th %	Mean	SD	Median	5th %	95th %
Young	3.4	2.1	3	0	7.35	265	230	175	30	809
Elderly	3.0	1.5	3	0	6	165	130	147	0	395

**Figure 5:**
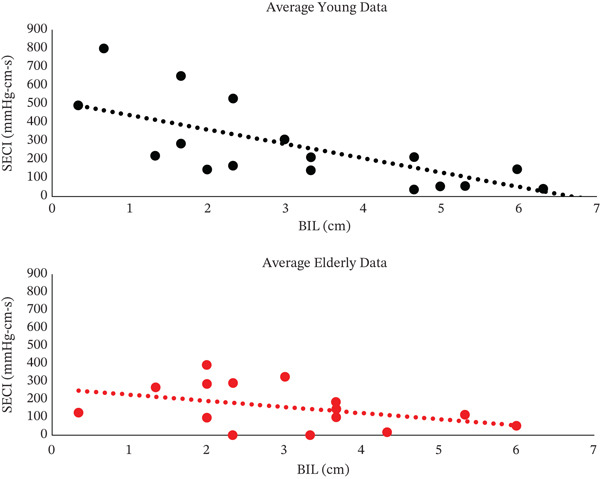
The interaction of striated esophageal flow dynamics and peristaltic contractile vigor illustrated by the correlation between BIL and SECI showed a significant correlation (*r* = −0.71, *p* = 0.0009) when comparing the average BIL to the average SECI in young subjects. This correlation was muted in the elderly and did not reach statistical significance (*r* = −0.41, *p* = 0.13).

The interaction of striated esophageal flow dynamics and peristaltic contractile vigor is illustrated by the correlation between BIL and SECI. As seen in Figure 5, a significant correlation (*r* = −0.71, *p* = 0.0009) was found when comparing the average BIL to the average SECI in young subjects. This correlation was muted in the elderly and did not reach statistical significance (*r* = −0.41, *p* = 0.13).

Further analysis was performed to discern differences in correlation between younger and older subjects. Multiple regression analyses showed the correlative relationship between BIL and SECI to be significantly different between young and elderly groups (*p* = 0.025).

## 4. Discussion

Transfer of the swallowed bolus from the pharynx into the esophagus is one of the critical events during swallowing due to proximity to the airway. In addition to clinical importance, it is a physiologically unique process in which a completely different neuromuscular contractile system, namely the oropharynx, seamlessly delivers the bolus to a differently controlled neuromuscular system, namely the StEso. Characterization of the functional correlation for a safe transorgan bolus transit has only recently become the topic of investigation.

In the present study, we have characterized the effects of aging on the correlation of the StEso deglutitive function with pharyngeal phase swallowing. Furthermore, we characterized and compared the inter‐relationship between BIL namely the distance that the pharyngeal phase swallow injects the bolus into the StEso before initiation of its peristalsis and the rigor of subsequent SECI among healthy young and elderly subjects. The study findings indicate that (a) overall correlation of StEso deglutitive motor function with pharyngeal phase of swallowing in the elderly is preserved compared with the young; (b) StEso deglutitive motor function spans both pharyngeal and esophageal phases of swallowing in both young and elderly; (c) the study findings also indicate the differences in the early period of spatiotemporal correlation of StEso deglutitive motor function with pharyngeal phase swallow biomechanics. These differences we believe can be most likely due to age‐related suprahyoid muscle weakness in the elderly [26, 49–51] since the affected periods are those associated with the active StEso ascent and descent, which are actualized by the contraction of these muscles; and (d) furthermore, concurrent manometry and video‐fluoroscopy indicates that HRM recordings currently attributed to the StEso deglutitive motor activity does not represent the entirety of StEso peristalsis, only the part that occurs in its resting position and contractions occurring during its descent are recorded as distal pharyngeal pressure/isocontour described previously [1, 2].

Additionally, our study findings suggest a negative correlation between the length of a liquid bolus projected into the StEso (BIL) before development of primary esophageal peristalsis and the contractile vigor of the ensuing primary esophageal peristaltic contraction. Although correlation does not necessarily mean causality, it is unlikely that the described correlation is merely coincidental and may suggest the distending effect of a long‐distributed bolus versus short‐distributed bolus on StEso wall mechanoreceptors. This negative correlation between the length of a liquid bolus injected into the StEso in period two and the vigor of the ensuing primary esophageal peristaltic contraction in period three which is indicative of a functional interplay between pharyngeal bolus propulsive forces and contractile vigor of the striated esophageal muscle was found to be muted in the elderly subjects tested in the present study. The mechanisms of the effect of aging on this relationship is not definitively known and require further studies but may be related to deterioration of the afferent sensory pathways of the feedback loop between the esophagus and the brainstem swallowing center and/or age‐related weakness of skeletal muscle of the proximal esophagus as part of general sarcopenia effect observed in the elderly [52, 53].

Although the overall correlation of deglutitive St. Eso motor function and the pharyngeal phase of swallowing is preserved in the elderly compared with the young, the observations reported in the present study carry similar ramifications for interpreting HRM data at the pharyngo‐esophageal junction as described in earlier publications [1]. These studies using concurrent HRM and digital video fluoroscopy showed that part of the isocontour in pharyngeal HRM recording that temporarily occurs following closure of the UES in fluoroscopy reflects the contraction of the descending StEso and closed UES and not the pharynx.

In conclusion, the overall correlation of StEso deglutitive motor function with the pharyngeal phase of swallowing in the elderly is preserved compared with the young. StEso deglutitive motor function in relation to the pharyngeal phase of swallowing shares similar attributes in elderly and young healthy subjects in that StEso function spans both pharyngeal and esophageal phases of swallowing. The differences observed in early stages of spatiotemporal mapping of StEso deglutitive motor function when comparing healthy elderly to young populations may be due to age‐related suprahyoid muscle weakness. The inter‐relationship of the length of the injected bolus into the StEso and StEso contractile vigor during ensuing peristalsis is weakened possibly due to age‐related neuromuscular deterioration. Like young subjects, the pressure signature in HRM recording currently attributed to the StEso deglutitive motor activity does not represent the entirety of StEso peristalsis, only the part that occurs in its resting position.

## Funding

This study was supported by the National Institute of Diabetes and Digestive and Kidney Diseases (10.13039/100000062, R01DK132082).

## Conflicts of Interest

The authors declare no conflicts of interest.

## Data Availability

The data that support the findings of this study are available from the corresponding author upon reasonable request.
